# Hemophagocytic Lymphohistiocytosis: A Case Report

**DOI:** 10.7759/cureus.61015

**Published:** 2024-05-24

**Authors:** Teresa Costa e Silva, Hugo Jorge Alves, João Horta Antunes, Carla Noronha, Bárbara Sousa Picado

**Affiliations:** 1 Internal Medicine, Hospital Beatriz Ângelo, Loures, PRT

**Keywords:** hemophagocytic lymphohistiocytosis, hemophagocytic syndrome, inflammatory syndrome, sepsis, drepanocytosis

## Abstract

The hemophagocytic syndrome (HS) or hemophagocytic lymphohistiocytosis (HLH) is a syndrome with apoptosis deficiency that results in the impairment of a regulatory pathway with consequent immune and inflammatory responses. Fever, cytopenias, splenomegaly, and hemophagocytosis are cardinal signs. It may be familial or secondary to infection, autoimmunity, or neoplasia. Impaired natural killer (NK)-cell cytotoxicity is the hallmark of HLH. All genetic defects in familial HLH are related to granule-dependent cytotoxicity.

The authors present a 50-year-old black female patient with a history of drepanocytosis who attended the emergency department due to fever, asthenia, lethargy, and hypogastric pain. Her laboratory workup on admission revealed severe pancytopenia. She was ultimately diagnosed with HLH due to sepsis of urinary origin, with a fatal outcome.

HLH is a rare and life-threatening syndrome. The delay in its diagnosis due to the variability of the clinical and laboratory findings constitutes the main obstacle to a successful prognosis, as illustrated in this case report.

## Introduction

Hemophagocytic syndrome (HS), also known as hemophagocytic lymphohistiocytosis (HLH), is a severe, self-sustaining inflammatory syndrome caused by an excessive, prolonged, and ineffective immune response [1]. HLH affects children more often than adults, namely infants with less than one year of life, due to a diversity of X-linked and autosomal recessive disorders. According to the etiology, HLH can be classified into genetic/primary and acquired/secondary types. Among primary HLH types, there is a further subdivision between familial HLH and other genetically associated forms of HLH. Secondary HLH can typically be triggered by infections, malignancies (mainly hematological, such as T-cell and natural killer-cell [NK] lymphomas), autoimmune diseases, and/or immune deficiency status [2].

The pathophysiology of HLH remains incompletely understood. However, cytotoxic stimulation of NK cells and/or T lymphocytes results in augmented serum cytokine levels and consequent multiplication of T cells and macrophages within target organs such as bone marrow, spleen, liver, and lymph nodes [3,4].

A definite consensus on the diagnostic criteria of HLH is still lacking. The clinical characteristics of HLH are extremely heterogeneous, yet the disease commonly presents with unremitting fever, splenomegaly, and peripheral blood cytopenias [5].

Adults with secondary HLH have extremely elevated mortality rates and a poor prognosis. Prompt recognition, diagnosis, and treatment of HLH are key to reversing a bad prognosis [2].

This case illustrates how important it is to have a high suspicion for HLH in patients with drepanocytosis who present with fever, pancytopenia, and multi-organ dysfunction that fail to respond to standard supportive therapy.

This article was previously presented as an oral communication at the 28th National Congress of Internal Medicine (CNMI) on October 2-5, 2022, and was distinguished as one of the top five oral communications.

## Case presentation

A 50-year-old black woman, bedridden due to a prior stroke, was admitted to the emergency department with a fever (38.5-39 °C) and asthenia with a one-week evolution. On the day of admission, the family also noticed lethargy, food refusal, and hypogastric pain. Besides a prior stroke with secondary epilepsy, her past history was significant for drepanocytosis with autosplenectomy, hypertension, mitral valve rheumatic disease, and pulmonary hypertension. Home therapy was hydroxyurea, amlodipine, levetiracetam, aspirin, and simvastatin. Physical examination revealed a lethargic, uncooperative patient with a fever (38.7 °C), hypotension (90/41 mmHg, assessed by the oscillometric method), and tachycardia (FC 130 bpm). She was pale and dehydrated, and her hypogastric palpation was painful yet without signs of peritoneal irritation. Neurological examination denied meningeal irritation and revealed sequelae of left hemiparesis.

Laboratory workup revealed a severe pancytopenia (hemoglobin 6.2g/dL, white blood cells 0.14/μL, neutrophils 0.01/μL, platelets 7000/μL), altered coagulation values (prothrombin time 15.1 seconds and activated partial thromboplastin time 32.1 seconds), renal dysfunction (blood urea nitrogen 65 mg/dL, creatinine 2.56 mg/dL), mild hypertransaminasemia (aspartate aminotransferase [AST] 145UI/L, alanine aminotransferase [ALT] 65UI/L), increased lactate dehydrogenase (LDH) (212 UI/L), and elevated inflammatory parameters (C-reactive protein 26.32 mg/dL and procalcitonin 75.8 ng/mL). No hyperlactatemia was found. A blood smear revealed rare target cells and hemoglobin S levels of 65%. An abdominopelvic computed tomography (CT) showed gastric distention with liquid content in pelvic and left flank locations, loops of small intestine of prominent caliber, slight perihepatic fluid, and voluminous global cardiomegaly. Urinalysis was pathologic, demonstrating leukocyte esterase and leukocyturia.

Volemic resuscitation using crystalloid fluids started with an unsatisfactory response, forcing the use of vasopressors. The patient was admitted to the ICU with the diagnosis of sepsis of urinary origin. Blood and urine cultures were collected, and empiric antibiotic therapy was established with meropenem due to a prior recent scheme of antibiotic therapy. Concurrent transfusion support was needed, namely red blood cells and platelet units. The use of prokinetics and a nasogastric tube helped in the amelioration of alimentary refuse.

After four days in the ICU, some improvement was noted (hemoglobin 8.2 g/dL, white blood cells 0.81/μL, and platelets 8000/μL). Moreover, *Escherichia coli* was identified both in hemocultures and urine culture, and *Proteus mirabilis* in urine culture. We performed a de-escalation from meropenem to cefuroxime, followed by a transfer to the Internal Medicine ward.

Three days after the transfer, there was a deterioration in the patient's clinical and laboratory conditions, primarily an aggravation of cytopenias, along with apyrexia and normal blood pressure.

**Figure 1:**
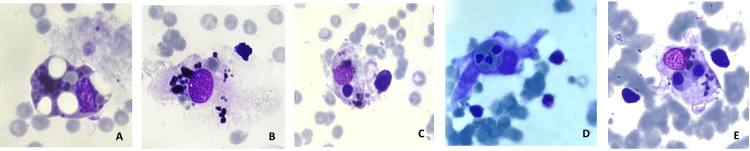
Bone marrow examination. (A) Histiocyte vacuolization; (B) Erythrocyte hemophagocytosis; (C) Lymphocyte hemophagocytosis; (D) Neutrophil hemophagocytosis; (E) Erythroblasts hemophagocytosis.

Therefore, bearing well in mind the patient’s clinical data, we performed serum ferritin concentration (3134 μL/L) and triglyceride count (3.47 mmol/L), focusing our suspicion on a macrophage activation syndrome, namely, a secondary form of HLH. A bone marrow examination was conducted, presenting macrophages with images of hemophagocytosis (Figure 1), Perl’s stain with greatly increased extra erythroblastic deposits of hemosiderin, and a bone marrow reactive to an inflammatory/infectious process. The bone marrow culture was negative.

Pulses of methylprednisolone (1 mg/kg/day) were introduced before the definitive result of the examination was known. Despite this treatment attempt, an unfavorable outcome was seen.

## Discussion

The clinical presentation of HLH is extremely varied. However, fever associated with multiple organ involvement/failure is a common disease feature. A definite consensus on diagnostic criteria is yet to be established, especially in adulthood. In 2004, the Histiocyte Society validated diagnostic criteria for children but not for adults, although they were commonly applied (Table 1) [6]. Later, in 2009, a new set of diagnostic criteria was proposed (Table 2) [7].

**Table 1:** Diagnostic criteria for hemophagocytic lymphohistiocytosis. Adapted from Henter et al. [6].

Diagnostic criteria
The diagnosis of hemophagocytic lymphohistiocytosis can be established if one of either 1 or 2 below is fulfilled:
1. A molecular diagnosis consistent with hemophagocytic lymphohistiocytosis
2. Diagnostic criteria for hemophagocytic lymphohistiocytosis fulfilled (5 out of the 8 criteria below)
Fever
Splenomegaly
Cytopenias (affecting ≥2 of 3 lineages in the peripheral blood)
Hemoglobin <9 g/dL
Neutrophils <1 × 10^9^/L
Platelets <100 × 10^9^/L
Hypertriglyceridemia and/or hypofibrinogenemia
Fasting triglycerides ≥3 mmol/L or ≥265 mg/dL
Fibrinogen ≤1.5 g/L
Hemophagocytosis in bone marrow, spleen, or lymph nodes
Absent or very decreased natural killer function
Ferritin ≥500 μg/L
Soluble interleukin-2 receptor ≥24,000 U/L

**Table 2:** Proposed hemophagocytic lymphohistiocytosis diagnostic criteria. Adapted from Filipovich [7].

Diagnostic criteria
1. Molecular diagnosis of hemophagocytic lymphohistiocytosis or X-linked lymphoproliferative syndrome
2. Or at least 3 of 4:
Fever
Splenomegaly
Cytopenias (minimum 2 cell lines reduced)
Hepatitis
3. And at least 1 of 4:
Hemophagocytosis
↑ Ferritin
↑ Soluble interleukin-2 receptor (age-based)
Absent or very decreased NK function
4. Other results supportive of HLH diagnosis:
Hypertriglyceridemia
Hypofibrinogenemia
Hyponatremia

The H score, proposed by Fardet et al., is a validated score designed for use in adult patients to estimate the likelihood of HLH in this population. This score integrates graded clinical and laboratory factors, including immunosuppression, fever, organomegaly, fibrinogen, ferritin, ALT, triglycerides’ level, the extent of cytopenias, and the existence of hemophagocytosis on the bone marrow aspirate. An H score of 250 or higher points confers a 99% probability of HLH, whereas a score of 90 or lower confers a probability of less than 1% of HLH [8,9].

Our patient met five out of eight criteria, namely, fever, peripheral blood cytopenia, hyperferritinemia, hypertriglyceridemia, and hemophagocytosis in the bone marrow. It should be noted that the autosplenectomy condition presented by the patient automatically made the splenomegaly criterion non-applicable. Concerning the H score, she scored 218 points, which corresponded to a 93-96% probability of HLH.

One of our patient's diagnostic dilemmas concerned the past history of sickle cell disease. The clinical and laboratory findings of sickle cell vaso-oclusive crises and HLH can partially overlap, making the diagnosis of HLH more challenging in these patients. HLH in association with drepanocytosis is not well characterized, and the majority of the sparsely described cases have been related to infection [10]. The other diagnostic dilemmas stemmed from the authors’ challenge in discerning whether the clinical presentation indicated a severe infection or HLH, with infection being the likely underlying cause. This issue arises because the clinical signs of HLH often resemble a variety of severe systemic conditions, such as septic shock, systemic inflammatory response syndrome, and multiple organ dysfunction syndrome, leading to significant diagnostic complications. Some experts suggest that sepsis and HLH form a disease *continuum* with a common underlying mechanism - systemic immune dysregulation triggered by a specific external agent [4,11]. Nevertheless, from a practical perspective, HLH should be considered in critically ill patients with fever of unknown origin, cytopenias, and organ dysfunction, especially in those non-responsive to aggressive sepsis therapy [12].

The absence of pain on admission or other clinical signs linked to a vast-occlusive crisis plus persistent pancytopenia, hemophagocytosis in the bone marrow, and, in a second stage, an unfavorable response to supportive therapy measures clinched the diagnosis of HLH.

Prompt treatment of HLH is vital. However, the main obstacle to achieving a positive outcome is the delayed diagnosis caused by its uniqueness, heterogeneous clinical features, and non-specificity and non-sensitivity of the clinical and laboratory findings.

Specific HLH treatment involves insistent suppression of the hyperinflammatory reaction and cytokine storm. Steroids are usually the hallmark of initial immunosuppression. If already prescribed in “sepsis-dose” (as in hydrocortisone), it may be switched to a regimen of high-dose pulsed methylprednisolone [13], as was performed in our patient. Other drugs, such as etoposide, and second- or subsequent-line treatment options include noncytotoxic immunochemotherapy agents [14].

In this patient, the initial clinical scenario pointed to sepsis as the primary diagnostic hypothesis. Moreover, the initial clinical and analytical improvement after the antibiotics’ prescription strongly contributed to the misconception of the correct diagnosis. Hyperferritinemia, persistence of cytopenias, and hypertriglyceridemia shifted the focus to a form of immune system dysregulation and led to the performance of bone marrow aspiration, which sadly came as late as corticosteroids, not allowing for targeted therapy before the patient passed away.

## Conclusions

HLH is a rare and life-threatening syndrome. The delay in its diagnosis, due to the variability of the clinical presentation and the insufficient specificity of the clinical and laboratory findings, constitutes the main obstacle to a successful prognosis, as unfortunately seen in the present clinical case. The broad pathogenic landscape of HLH, along with its high mortality rate, makes this disease one of the most complex disorders to manage. The development of evidence-based guidelines and novel therapies is challenging yet necessary for better outcomes in HLH.
